# High-dose immunoglobulin-dependent chronic demyelinating inflammatory polyneuropathy successfully managed with subcutaneous immunoglobulin using pharmacokinetic analysis

**DOI:** 10.1016/j.ensci.2022.100404

**Published:** 2022-05-11

**Authors:** Satomi Hiya, Satoru Fujiwara, Fumiaki Tanaka, Nobuo Kohara, Michi Kawamoto

**Affiliations:** a Department of Neurology, Kobe City Medical Center General Hospital, Kobe, Hyogo, Japan; b Department of Pharmacy, Kobe City Medical Center General Hospital, Kobe, Hyogo, Japan

**Keywords:** Chronic inflammatory demyelinating polyradiculoneuropathy, Pharmacokinetic analysis, Autoantibodies, Diffuse demyelination, Immunoglobin G, Intravenous immunoglobulin, Subcutaneous immunoglobulin

## Abstract

Immunoglobulin G therapy for chronic inflammatory demyelinating polyneuropathy (CIDP) often requires individual dose adjustments because of the heterogeneity of pathogenesis and varying catabolic rates. However, currently available pharmacokinetic studies of immunoglobulin G therapy do not consider individual differences. We conducted a pharmacokinetic study of both intravenous immunoglobulin and subcutaneous immunoglobulin in a single patient with CIDP who was dependent on high-dose immunoglobulin treatment. This patient—a 77-year-old man with symmetrical limb weakness, diffuse demyelination determined by a nerve conduction study, and lacking autoantibodies—was treated with intravenous immunoglobulin and experienced severe fluctuations in symptoms. We transitioned him to subcutaneous immunoglobulin: his serum immunoglobulin G levels stabilised and he experienced symptomatic relief. Monitoring of serum immunoglobulin G concentrations revealed volatile changes following intravenous immunoglobulin administration which stabilised following subcutaneous immunoglobulin treatment. This suggests that subcutaneous immunoglobulin is a preferable long-term treatment option, especially for high-dose immunoglobulin-dependent patients with CIDP.

## Highlights

• When the target serum IgG level is high, IVIg accelerates IgG catabolism and causes volatile changes in serum IgG levels.
• SCIg can promote a stable serum IgG concentration with the same monthly dosage as IVIg treatment, without accelerating catabolism. This may improve clinical outcomes.
• SCIg might be a successful treatment option for other patients with CIDP whose symptoms are uncontrolled or sub-optimally controlled by high-dose IVIg.

## 1 Introduction

Clinicians have long recognised immunoglobulin G (IgG) as a first-line therapeutic for chronic inflammatory demyelinating polyradiculoneuropathy (CIDP). Although guidelines suggest standard doses for administering IgG intravenously or subcutaneously (IVIg and SCIg, respectively), [1] individual patients often require unique adjustments to their doses and intervals between doses. [2] Some patients with CIDP have reportedly only shown improvement when they have taken a high dose of IgG. [3] The aetiological heterogeneity of CIDP contributes to such patient-to-patient differences in response to IgG therapy. [4] Furthermore, the catabolic rate of IgG differs considerably among individuals, [2] highlighting the importance of pharmacokinetic studies in deciding the therapeutic dose of IgG for individual patients. The few currently available pharmacokinetic studies on IgG treatment for CIDP do not consider individual differences and do not address the challenges of treating high-dose-dependent patients. Thus, there is inadequate information about treatment strategies for such subgroups of patients with CIDP. Therefore, we conducted this pharmacokinetic study of IgG administration in a single CIDP patient with high-dose IgG dependency.

## 2 Case

A 77-year-old man with a history of prostate cancer developed weakness of the left leg followed by dysarthria, both of which resolved within 2 months. A month later, he developed weakness of the left leg that extended to both arms and legs. The patient was unable to walk and was hospitalised. His symptoms resolved after 2.0 g/kg IVIg was administered over 5 days. However, a month later, the limb weakness recurred. Steroids were administered; however, the patient showed no improvement.

The patient was referred to our institution for further treatment. On admission, he was unable to lift any of the extremities against gravity. The patient experienced relatively minor sensory disturbance which was restricted to the palm and sole. There were no deep-tendon reflexes. We observed mild dysphagia and dysarthria. The cerebrospinal fluid analysis results were within normal limits (proteins: 35 mg/dL; cells: 1/μL; glucose: 82 mg/dL) and showed normal opening pressure. Laboratory test results for complete blood count, erythrocyte sedimentation rate, biochemical profile, and thyroid function tests, were within normal ranges. The results of serum and urine immunoelectrophoresis and serological tests for infectious diseases and levels of vitamins, blood glucose, HbA1c, angiotensin-converting enzyme, and anti-neutrophil cytoplasmic antibodies were unremarkable. Serum levels of rheumatoid factor and double-stranded DNA were also negative. The anti-nuclear antibody level was 40 times above the normal level, and the anti-SS-A antibody titre was 17.2 U/mL. The serum immunoelectrophoresis test was negative for monoclonal gammaglobulins. Serum anti-contactin 1 and anti-neurofascin 155 antibodies were proved negative by enzyme-linked immunosorbent assay. A nerve conduction examination revealed delayed distal latency, decreased motor nerve conduction velocity, and temporal dispersion in compound motor action potential on the median, ulnar, peroneal, and tibial nerves bilaterally. The amplitude of sensory nerve action potential was decreased on the median, ulnar, and tibial nerves bilaterally and was relatively normal on both sural nerves (Table 1). These findings met the electrodiagnostic criteria for definite CIDP according to the European Academy of Neurology/Peripheral Nerve Society, [1] and we confirmed a diagnosis of motor CIDP without autoantibodies.

**Table 1:** The nerve conduction study. CMAP: Compound muscle action potential, SNAP: Sensory nerve action potential, Lat.: Latency, Amp.: Amplitude, Dur.: Duration, NCV: Nerve conduction velocity.

	Right				Left			
	Lat. (ms)	Amp. (mV)	Dur. (ms)	NCV (m/s)	Lat. (ms)	Amp. (mV)	Dur. (ms)	NCV (m/s)
CMAP								
Median								
Wrist	8.04	2.4	7.65		7.55	2.95	15.15	
Elbow	17.91	0.76	9.69	22.3	16.25	1.27	15.30	21.2
Ulnar								
Wrist	4.86	4.04	8.34		3.95	4.31	7.80	
Below Elbow	9.51	2.50	9.30	43.0	11.05	3.52	9.10	31.0
Peroneal								
Ankle	4.74	1.65	7.17		7.80	0.88	8.20	
Fibular head	13.5	1.23	8.40	35.4	16.85	0.81	11.0	33.1
Tibial								
Ankle	5.34	8.95	13.17		6.50	5.61	25.25	
Popliteal	14.55	6.83	15.24	38.5	16.6	5.74	20.4	36.6

SNAP								
Median								
Wrist	3.26	4.50	1.98	41.7	3.14	7.40	2.42	48.6
Elbow	7.62	2.70	3.82	50.5	9.08	1.60	2.52	40.06
Ulnar								
Wrist	3.00	5.90	2.90	48.3	3.24	4.00	2.58	35.5
Below Elbow	7.08	2.80	3.30	47.8	8.42	1.50	2.94	42.5
Sural	2.52	9.50	2.52	55.6	3.38	8.70	1.88	41.4

## 3 Clinical course

We first administered 2.0 g/kg IVIg over 5 days. The patient's muscle strength improved as the serum IgG level increased. However, several days after his IgG level decreased, he gradually lost muscle strength, suggesting IgG dependency. We initiated a maintenance infusion of 0.4 to 1 g/kg/week depending on the patient's muscle strength. We added cyclosporin A to reduce IgG dependency. Nine weeks after admission, the patient was able to walk without assistance. We discharged him from hospital with a planned maintenance dosage of 0.5 g/kg IVIg every 2 weeks. The serum IgG level at discharge was 1504 mg/dL.

**Fig. 1:**
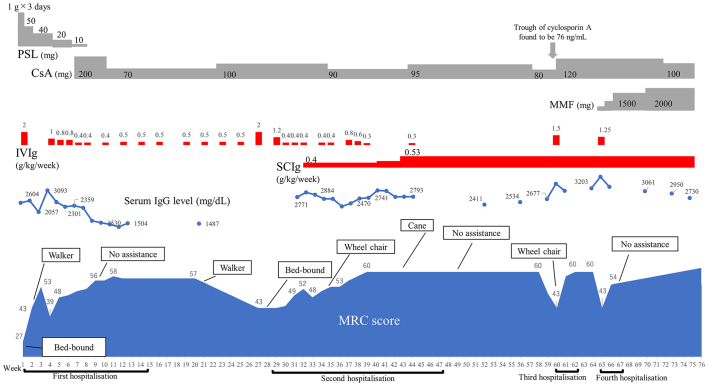
Treatment, serum IgG level and symptom response. The patient showed IgG dependency. Both deterioration and recovery followed after IgG level decrease and increase with some delay. The patient achieved symptom stability with subcutaneous immunoglobulin use but experienced two less-severe relapses. PSL: prednisolone, CsA: cyclosporin A, MMF: mycophenol mofetil, IVIg: intravenous immunoglobulin, SCIg: subcutaneous immunoglobulin, MRC: medical research council.

Several weeks after discharge, our patient gradually lost muscle strength, was bedridden again, and returned to our institution at week 28. We set the target serum IgG concentration at 2000 to 3000 mg/dL, the level at which the patient's muscle strength was consistently recovered during the first hospitalisation. To achieve a stable IgG concentration, we decided to introduce SCIg during the second admission. After administering rescue IVIg infusions, we initiated a SCIg dosage of 0.4 g/kg/week at week 33, increasing it later to 0.53 g/kg/week to prevent symptom fluctuations. We measured serum IgG at least once during the second hospitalisation to compare the serum IgG level and the symptom. The patient's serum IgG concentration was stable between 2500 and 3000 mg/dL when we discharged him again (Fig. 1).

## 4 Pharmacokinetic study

We noticed rapid changes in the patient's serum IgG levels, especially during maintenance therapy with IVIg. We conducted pharmacokinetic analyses of IVIg and SCIg administration to estimate the patient's IgG catabolism limitations using NONMEM software (version 7.4.1; Icon Development Solutions, Ellicott City, MD, USA). We estimated the pharmacokinetic limits for volume of distribution (Vd) and clearance using the Bayesian method based on a previously reported population pharmacokinetic model. [5,6] The elimination rate of IgG varied after each dose of IVIg administration. To better approximate the patient's measured IgG level by the simulated value, we subdivided the whole IVIg treatment period into 13 short periods, estimating Vd and clearance for each period to simulate the curve of the serum IgG level. We also calculated the elimination constant (Ke) and half-life for each short period as Ke = clearance/Vd and half-life = (loge2÷24)/Ke using the estimated values of Vd and clearance. The mean half-life of IgG during the period of IVIg and SCIg administration was 19.0 ± 5.81 and 42.7 days, respectively (Table 2). Thus, if different population models are used, the half-life may change.

**Table 2:** Simulated Vd, Half-life in IVIg-only period and SCIg-only period. The whole IVIg period was divided into 13 short periods and SCIg period into 3. Vd: volume of distribution, T1/2: half-life. Clearance and Vd are obtained by Bayesian inference using NONMEM software. Elimination constant (Ke) and T1/2 are calculated by: Ke = CL/Vd, T1/2 = (loge2÷24)/Ke.

Week	Vd(L) (Central and peripheral compartments)	T1/2 (day)
IVIg period		
0〜2.1	4.75	12.3
2.1〜3.1	4.43	20.7
3.1〜4.5	3.87	12.5
4.5〜5.4	4.88	20.1
5.4〜6.4	5.13	18.3
6.4〜7.0	4.35	17.9
7.0〜9.1	4.28	14.1
9.1〜10.1	4.93	20.5
10.1〜11.1	4.80	21.5
11.1〜28.9	5.83	34.3
28.9〜30.0	5.56	20.3
30.0〜31.3	4.82	21.4
31.3〜33.3	4.07	13.0
Mean (±SD)	4.75(±0.56)	19.0(±5.81)

SCIg period		
67.2–76.3	9.08	42.7

**Fig. 2:**
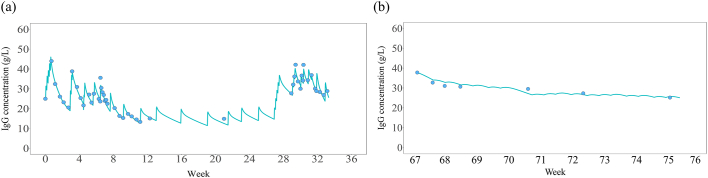
Simulated change of serum IgG levels.

The results demonstrated that the patient's IgG level fluctuated significantly and rapidly when he was on IVIg therapy, but remained stable after transitioning to SCIg. There was high volatility in serum IgG levels at high serum concentrations during IVIg treatment (Fig. 2).

After transitioning to SCIg, our patient developed two episodes of mild relapse, including complaint of weakness of the hands and difficulty in standing up from a sitting position. We managed each relapse by increasing the dosage of cyclosporin and adding mycophenolate mofetil. We maintained the serum IgG level at between 2500 and 3000 mg/dL, and the patient followed an uneventful course.

## 5 Discussion

A previous study reported that a subgroup of CIDP patients develops an exceptionally high dependency on IgG treatment. [3] The clinical characteristics of such patients include motor CIDP, symmetrical weakness, and diffuse conduction abnormalities detected by nerve conduction studies, and resistance to steroids, plasma exchange, and immunosuppressants. Another study of four patients described optimum serum IgG levels of 2900–3400 mg/dL: patients required monthly immunoglobulin dosages of 2.7–4.4 g/kg. [3] Our patient demonstrated clinical features similar to those patients, suggesting that they all belong to the same CIDP subgroup. Our patient's optimum IgG level was 2500–3000 mg/dL. We administered SCIg twice a week to achieve a monthly cumulative dosage of 2.13 g/kg. Our patient demonstrated severe symptom fluctuations during IVIg therapy but maintained symptom stability during SCIg therapy. Clinical observations and pharmacokinetic studies suggested that high-dose IVIg could not prevent rapid changes in serum IgG levels because of accelerated IgG catabolism, but SCIg could maintain a stable IgG concentration and eventually led to lasting symptom relief.

Our patient demonstrated a relatively fast catabolic rate of IgG. The half-life of IgG is generally accepted to be between 21 and 30 days, while his was only 19 days. [7] Our patient may have had fast catabolism for inherent reasons such as low neonatal Fc receptor activity [8] or because of the concentration-dependent acceleration of IgG catabolism, as documented previously. [7] In our simulation, we observed a relatively short half-life of 12 to 13 days when serum IgG levels were especially high (Fig. 2 and Table 2). This suggests that when managed with IVIg, a high target serum IgG expected to exert a more positive effect can paradoxically increase the catabolic rate of IgG. Hence, we would need to maintain the target concentration through high dosage and frequency. Our simulation showed that more frequent dosing, such as twice a week, failed to prevent the rapid drop in IgG levels that made symptom stabilisation difficult.

After a complete transition to SCIg, our patient's serum IgG level stabilised and symptom fluctuation reduced. Many SCIg studies have reported on the slow absorption achieved using the subcutaneous route. [9] IVIg causes a volatility in serum levels, primarily because of its increased dosage; this eventually leads to accelerated catabolism. SCIg can maintain stable serum IgG levels with the same monthly cumulative dosage. The half-life of SCIg in our patient was 43.1 ± 5.6 days, which is close to the mean of the reference population. [6] This suggests that SCIg does not accelerate catabolism, even at high dosage. It may then be especially beneficial for patients who show accelerated IVIg catabolism regardless of whether the catabolism is intrinsically fast or accelerated because of high dosage.

About 9% of all CIDP cases are of the motor CIDP. These patients reportedly have a response rate of approximately 82% to IVIg treatment, [4] and may be maintained on IVIg therapy with considerable symptom fluctuations. They might achieve better treatment response rates and be more stable on high-dose SCIg therapy. This can eventually reduce unnecessary use of immunosuppressants and other invasive and expensive treatments.

## Funding

This research did not receive any specific grant from funding agencies in the public, commercial, or non-profit sectors.

The peaks of the blue line are considered a consequence of immunoglobulin intravenous infusion (left) or subcutaneous injection (right). Blue dots represent actually measured IgG level. (a) Changes in serum IgG level when the patient was managed with IVIg only (from week 0 to week 36 in Fig. 1) are volatile with high peaks and low troughs, even with frequent infusions. (b) The smooth line with almost no obvious peaks and troughs show the stabilised IgG levels.

## Declaration of Competing Interest

The authors report no conflict of interest.

## References

[1] Van den Bergh P.Y.K., van Doorn P.A., Hadden R.D.M. (2021). European academy of neurology/peripheral nerve society guideline on diagnosis and treatment of chronic inflammatory demyelinating polyradiculoneuropathy: report of a joint task force-second revision. J. Peripher. Nerv. Syst..

[2] Allen J.A., Berger M., Querol L. (2018). Individualized immunoglobulin therapy in chronic immune-mediated peripheral neuropathies. J. Peripher. Nerv. Syst..

[3] Debs R., Reach P., Cret C. (2017). A new treatment regimen with high-dose and fractioned immunoglobulin in a special subgroup of severe and dependent CIDP patients. Int. J. Neurosci..

[4] Doneddu P.E., Cocito D., Manganelli F. (2019). Atypical CIDP: diagnostic criteria, progression and treatment response. Data from the Italian CIDP database. J. Neurol. Neurosurg. Psychiatry.

[5] Tortorici M.A., Lawo J.P., Weide R. (2019). Privigen® has similar pharmacokinetic properties in primary and secondary immune deficiency. Int. Immunopharmacol..

[6] Zhang Y., Baheti G., Chapdelaine H. (2020). Population pharmacokinetic analysis of weekly and biweekly IgPro20 (Hizentra®) dosing in patients with primary immunodeficiency. Int. Immunopharmacol..

[7] Waldmann T.A., Strober W. (1969). Metabolism of immunoglobulins. Prog. Allergy.

[8] Dalakas M.C., Spaeth P.J. (2021). The importance of FcRn in neuro-immunotherapies: from IgG catabolism, FCGRT gene polymorphisms, IVIg dosing and efficiency to specific FcRn inhibitors. Ther. Adv. Neurol. Disord..

[9] Wasserman R.L., Melamed I., Nelson R.P. (2011). Pharmacokinetics of subcutaneous IgPro20 in patients with primary immunodeficiency. Clin. Pharmacokinet..

